# The Blind

**Published:** 1886-10-02

**Authors:** John Milton


### THE BLIND.

These eyes, though clear
To outward view of blemish or of spot,
Bereft of light, their seeing have forgot;
Nor to their idle orbs doth sight appear
Of sun or moon or star throughout the year,
Of man or woman. Yet I argue not
'Gainst heaven's hand or will, nor bate a jot
Of heart or hope; but still bear up and steer
Right onward.

John Milton.

Dinners.—Dr. William Hunter, brother of the great anatomist, used never to regale himself with more than two dishes. He would say, "A man who cannot dine on this, deserves to have no dinner."

"Sir John."—There is a story told that John Hunter, the distinguished anatomist and surgeon, was once offered the honour of knighthood, but declined it, saying he was a *Sur-geon* already.

A physician's wife, who was bored with visitors, remarked to her husband at the table, where a number of self-invited guests were seated, "My dear, I was afraid the children would get hold of that leg you brought home from the dissecting-room last night, so I took it downstairs with me, and that's it wrapped up in a towel on the sideboard there." She dines pretty often without company now.

